# Circulating Extracellular Vesicles and Endothelial Damage in Sickle Cell Disease

**DOI:** 10.3389/fphys.2020.01063

**Published:** 2020-09-03

**Authors:** Gabrielle Lapping-Carr, Joanna Gemel, Yifan Mao, Eric C. Beyer

**Affiliations:** Department of Pediatrics, The University of Chicago, Chicago, IL, United States

**Keywords:** sickle cell disease, extracellular vesicle, exosomes, microvesicle, endothelial damage

## Abstract

Endothelial damage is central to the pathogenesis of many of the complications of sickle cell disease. Circulating extracellular vesicles (EVs) have been implicated in modulating endothelial behavior in a variety of different, diseases with vascular pathologies. As seen in other hemolytic diseases, the plasma of sickle cell patients contains EVs of different sizes and cellular sources. The medium-sized vesicles (microparticles) primarily derive from mature red blood cells and platelets; some of these EVs have procoagulant properties, while others stimulate inflammation or endothelial adhesiveness. Most of the small EVs (including exosomes) derive from erythrocytes and erythrocyte precursors, but some also originate from platelets, white blood cells, and endothelial cells. These small EVs may alter the behavior of target cells by delivering cargo including proteins and nucleic acids. Studies in model systems implicate small EVs in promoting vaso-occlusion and disruption of endothelial integrity. Thus, both medium and small EVs may contribute to the increased endothelial damage in sickle cell disease. Development of a detailed understanding of the composition and roles of circulating EVs represents a promising approach toward novel predictive diagnostics and therapeutic approaches in sickle cell disease.

## Endothelial Cells in the Pathogenesis of Sickle Cell Disease

Sickle cell disease (SCD) is a debilitating disorder in which a single amino acid substitution (Glu6→Val in β-globin) results in an abnormal hemoglobin with a propensity to polymerize and deform erythrocytes. The altered erythrocytes hemolyze easily, and their rigidity and abnormal shapes cause intermittent occlusion of the microvasculature. Repeated ischemic insults and ischemia/reperfusion injuries culminate in significant damage to many different organs, including the bones, lungs, brain, heart, kidneys, skin, spleen, and endocrine glands.

Alterations of the endothelium (including activation and damage) are central to the pathophysiology of sickle cell disease complications (Sundd et al., 2019). Sickle erythrocytes exhibit better adhesion to endothelial cells than red cells from normal individuals (Hoover et al., 1979; Hebbel et al., 1980). This increased endothelial adhesiveness reflects the increased expression or exposure of various adhesion molecules, including phosphatidylserine, the selectins (P- and E-), integrins, intercellular-adhesion-molecules (ICAM-1 and ICAM-4), and vascular-cell-adhesion-molecule-1. Recurrent episodes of occlusion cause direct endothelial injury due to ischemia and ischemia/reperfusion (Hebbel, 2014; Hebbel et al., 2020). The release of erythrocyte contents (like hemoglobin or heme) due to hemolysis cause endothelial activation through several pathways, including those driven by oxidative stress, Toll-like receptors, and NF-κB (Kato et al., 2017). Sickled red cells and the byproducts of hemolysis activate other blood cells (leukocytes and platelets). Endothelial cells and white blood cells release various cytokines. Studies in SCD mice have shown that some of these cytokines and other inflammatory mediators (like TNFα, heme, and lipopolysaccharide) can induce vaso-occlusion. Together, all of these factors create an altered inflammatory milieu in SCD patients (Hoppe, 2014).

There is significant variability in the severity and frequency of complications among individuals with SCD. Predicting complications and targeting therapies remain very challenging despite extensive investigations of the pathologic pathways. We speculate that differences in circulating extracellular vesicles (EVs) and their effects on endothelial cells may contribute to inter-patient variabilities in disease severity.

## Different Kinds of Extracellular Vesicles

EVs are small vesicles containing cellular contents surrounded by lipid bilayers that are produced by many different kinds of cells. EVs are best classified according to their differing cellular generation pathways, which correlate with their sizes. Large and medium EVs are created by cellular damage, while small EVs are actively secreted (Colombo et al., 2014; Cocucci and Meldolesi, 2015; Meldolesi, 2018). (1) Apoptotic bodies (800 nm–5 μm) are produced during apoptosis *via* membrane disintegration. (2) Microvesicles (200–1,000 nm) are formed by pinching off from the cell membrane; vesicles in this category have sometimes been termed ectosomes or microparticles. Microvesicles contain mainly cytosolic and plasma membrane associated proteins. (3) Small EVs (50–200 nm), which are often referred to as exosomes, are generated by release from the endosomal sorting complex required for transport (ESCRT). Therefore, ESCRT proteins and accessory proteins (Alix, TSG101, HSC70, and HSP90β) are found in exosomes, as are tetraspanins, including CD63, CD9, and CD81. Small EVs also contain nucleic acids, including mRNAs and miRNAs.

Many EVs end up within the bloodstream. When circulating EVs encounter endothelial cells, they can affect their behavior, either through interactions at the cell surface or by transfer of contents (including proteins, lipids, DNA, mRNA, and microRNAs) carrying signals from their cell of origin. Therefore, EVs (particularly exosomes, which contain nucleic acids) are thought to play a role in regulating endothelial responses to damage (Ridger et al., 2017; Oggero et al., 2019).

The International Society for Extracellular Vesicles (ISEV) has developed recommendations regarding collection of blood or culture samples, isolation of vesicles, and definition of the cellular origins of EVs (Théry et al., 2018). Methods used to isolate EVs include serial centrifugation, size exclusion chromatography, and precipitation. These techniques differ in the yield of EVs and their contamination with non-EV material. Depending on isolation methods, small EVs can be contaminated with clumps of proteins, viral particles, lipoproteins, and ectosomes. Differences in preparations due to differing isolation methods may contribute to confounding results in different studies.

## Extracellular Vesicles in the Pathogenesis of Vascular Diseases

The potential importance of EVs in contributing to the vascular abnormalities of SCD is supported by the data implicating them in other cardiovascular diseases that have some of the features observed in SCD. As with SCD, thrombosis, endothelial dysfunction and damage, ischemia-reperfusion injury, and inflammation are key components of various cardiovascular diseases.

Most of the links between circulating EVs and cardiovascular disease are correlative. Several studies have shown significantly increased levels of circulating EVs (microvesicles and exosomes) derived from endothelial cells, leukocytes, platelets, and/or erythrocytes in patients with different cardiovascular diseases, including ischemic coronary artery disease (reviewed by Jansen et al., 2017; Ridger et al., 2017). In diabetic patients, increased numbers of endothelial EVs correlate with endothelial dysfunction detected as worsened arterial elasticity and endothelium-dependent dilation (Feng et al., 2010).

Because vesicle composition and content represent specific signatures of cellular activation and injury, differences in EV profiles have been proposed as useful tools for diagnosing and monitoring risk and activity of cardiovascular diseases. Some studies have identified vesicular microRNAs that may discriminate different groups of patients (Jansen et al., 2017). As examples, in one study, increased levels of miR-126 and miR-199a in circulating EVs were associated with a lower rate of major adverse cardiovascular events (Jansen et al., 2014), while in a different study, patients with acute coronary syndrome had higher levels of miR-208a in serum EVs than control subjects (Bi et al., 2015).

The direct contribution of EVs to endothelial pathology is best supported by several examples of studies showing vascular and inflammatory effects of these EVs. The platelet microparticles may make a procoagulant contribution to thrombotic cardiac diseases through the exposure of negatively charged phosphatidylserine on their surfaces, which can enhance clot formation. Platelet microparticles also stimulate cultured endothelial cells to increase their adhesiveness (Barry et al., 1998). *In vitro*, endothelial EVs induce vasorelaxation and nitric oxide production, but they impair capillary angiogenesis (Brodsky et al., 2004; Mezentsev et al., 2005). Monocyte exosomes induce endothelial expression of cytokines and adhesion proteins (Tang et al., 2016).

There are also ongoing attempts to develop EVs as therapeutics for cardiovascular diseases. In stroke and myocardial infarction models, EVs from mesenchymal stem cells have increased survival of cardiomyocytes, promoted angiogenesis, decreased infarct size, and improved neurologic recovery (Giebel et al., 2017).

## Extracellular Vesicles in Other Hematologic Disorders

One of the major sequelae of SCD is hemolysis, the breakdown of erythrocytes. A predictable consequence of hemolysis is the generation of EVs derived from the red cells. Some insights regarding EVs in SCD can be garnered from studies of EVs in relation to other red blood cell disorders.

Generation and release of EVs occur during the normal maturation of red blood cells and during pathological damage. Secretion of exosomes is a major process by which maturing erythrocyte precursors (especially reticulocytes) dispose of membrane and cytosolic proteins (like transferrin receptor; Johnstone et al., 1987; Ovchynnikova et al., 2018). Microvesicles/ectosomes are generated by the outward budding from the plasma membrane of normal red blood cells (Nguyen et al., 2016). It is well-known to experts in blood banking that microvesicles are increasingly generated during storage of red blood cells (Kuo et al., 2017). These artificially generated EVs have been reported to have vasoregulatory and immunomodulatory properties (Said and Doctor, 2017; Almizraq et al., 2018). Microvesicles may also be generated from circulating erythrocytes by mechanical or complement-mediated damage (Westerman and Porter, 2016).

It has long been recognized that normal platelets and megakaryocytes also release medium-sized EVs (or microparticles; Crawford, 1971). The release of platelet microparticles can be triggered by activation of platelets by physiological agonists and by storage, cryopreservation, and shear stress (Boilard et al., 2015). These microparticles may have a procoagulant effect by providing a surface for coagulation factors to assemble (Owens and Mackman, 2011). Elevated levels of circulating microparticles have been observed in many thrombotic disorders (Nomura and Shimizu, 2015).

Like SCD, the thalassemias (a group of genetic disorders that involve underproduction of hemoglobin) are hemoglobinopathies that result in hemolysis (among other manifestations). However, much less endothelial damage occurs in the thalassemias than is typically observed in SCD. Several studies of β-thalassemia patients have demonstrated increased abundances of circulating medium-sized EVs, especially those who have been splenectomized (Agouti et al., 2015; Klaihmon et al., 2017a). These microparticles derive from erythrocytes, platelets, and endothelial cells. The platelet-derived microparticles have been implicated in the increased propensity of thalassemia patients to thrombosis (Pattanapanyasat et al., 2007; Agouti et al., 2015). The abundance of circulating EVs and the thrombotic risk return to normal after successful treatment of the thalassemia by hematopoietic stem cell transplantation (Klaihmon et al., 2017b).

Paroxysmal nocturnal hemoglobinuria (PNH) is a disease that results in hemolysis (and thrombosis) due to an acquired clonal abnormality that causes increased sensitivity to complement-mediated cell lysis. In PNH patients, the blood contains increased numbers of platelet EVs, while erythrocyte-derived EVs are present, but in quantities that are similar to those in control subjects without PNH (Ninomiya et al., 1999; Kozuma et al., 2011; Freitas Leal et al., 2019). However, in these patients, both groups of EVs appear to be prothrombotic (Kozuma et al., 2011). Interestingly, despite a lack of increased numbers, the vesicles that are present are clearly a degree more prothrombotic *in vitro* than in controls. Treatment of patients with Eculizumab (to block complement-mediated lysis) decreases both the abundance of microvesicles and the thrombotic risk (Weitz et al., 2012). *In vitro* studies related to PNH have demonstrated that microparticles can interact with erythrocytes, since microvesicles derived from normal red blood cells can transfer glycophosphoinositide-anchored proteins to PNH erythrocytes (Sloand et al., 1998). Only a few exploratory studies have examined small EVs in PNH (Teruel-Montoya et al., 2019; Vallejo et al., 2019).

Increased numbers of circulating microvesicles have also been detected in other hemolytic disorders, including autoimmune hemolytic anemia (Kidd et al., 2015), complement-mediated hemolysis (Arvidsson et al., 2015), malaria (Mantel et al., 2013), and hereditary erythrocyte membrane disorders (Alaarg et al., 2013). In contrast, reduced numbers of circulating microvesicles and external exposure of phosphatidylserine are observed in patients with Scott syndrome, a rare bleeding disorder of unknown genetic basis in which cells have abnormalities of cellular calcium handling (Bevers et al., 1992; Morel et al., 2011). Elucidation of this disease might help with the development of therapeutic approaches to reducing levels of procoagulant microparticles.

## Medium-Sized Extracellular Vesicles (Microparticles) in Sickle Cell Disease

There have been a number of studies of medium-sized EVs in relation to SCD (reviewed by Hebbel and Key, 2016). Many years ago, it was demonstrated that sickling and un-sickling of sickle erythrocytes induced by oxygenation-deoxygenation cause loss of membrane by the shedding of “micro-spherules” (Padilla et al., 1973) or the formation of “microspicules” that degrade to form microvesicles (Allan et al., 1982). These particles are spectrin-free, but their membranes are otherwise similar to the plasma membrane of red blood cells. They have red cell cytoplasmic contents like hemoglobin. Electron micrographs show that these microvesicles are rather heterogeneous with diameters of 150–400 nm (Allan et al., 1981).

These erythrocyte-derived microvesicles have been identified in the blood of sickle cell patients, as have vesicles derived from platelets, white blood cells, and endothelial cells (Tantawy et al., 2013; Hebbel and Key, 2016). The consensus of many studies is that the abundance of circulating microparticles is increased in individuals with SCD as compared with normal subjects, but the abundance is not consistently altered in association with pain crises (Nebor et al., 2014; Hebbel and Key, 2016). Microparticle levels are increased in association with hemolysis (Westerman et al., 2008; van Beers et al., 2009; Merle et al., 2018; Olatunya et al., 2019). It is unclear whether levels of microparticles (either total or those from a specific cell type) are useful biomarkers for disease severity or complications (Hebbel and Key, 2016).

Several studies have suggested possible pathogenic roles of circulating microparticles in SCD. Many of the microparticles contain tissue factor and may exert procoagulant effects (Shet et al., 2003; van Beers et al., 2009). The infusion of SCD erythrocyte microparticles in mice leads to production of reactive-oxidative species, vasodilation, and vaso-occlusion in the kidneys (Camus et al., 2015). These changes may be caused by transfer of heme contained in microparticles to endothelial cells. Microparticles released during vaso-occlusive crises increase endothelial ICAM-1 levels and neutrophil adhesion (Garnier et al., 2020).

A recent study emphasizes the importance of platelet EVs in the pathogenesis of SCD. Using sickle mice and human SCD blood flowing through a microfluidics chamber, Vats et al. (2020) found that activation of the platelet inflammasome led to the production of platelet EVs containing interleukin-1β and caspase-1. Moreover, the EVs promoted lung vaso-occlusion. Size analysis of the EVs in this study suggests that they included both small and medium-sized EVs.

Some treatments that have beneficial consequences for SCD patients may also modulate microparticle abundance or detrimental properties. Hydroxyurea treatment has been associated with a decrease in microparticles derived from erythrocytes and platelets (Nébor et al., 2013). Exchange transfusion may reduce the abundance of erythrocyte-derived microparticles (Mahfoudhi et al., 2012).

## Small Extracellular Vesicles in Sickle Cell Disease

There have only been a few studies of small EVs, or exosomes, in relation to the pathophysiology of SCD. We have shown that the plasma of children and young adults with SCD contains abundant small EVs (Khalyfa et al., 2016; Lapping-Carr et al., 2017). Small EVs can also be generated from the platelets of human SCD subjects and sickle mice (Vats et al., 2020).

The small EVs in our preparations comprise relatively uniform populations of particles as assessed by nanoparticle tracking analysis. Immunoblotting shows that the small EVs contain flotillin and CD63 (which are found in exosomes), but they do not contain proteins from the endoplasmic reticulum or lipoproteins (Lapping-Carr et al., 2020). In both control and SCD subjects, most of the circulating EVs derive from red blood cell precursors (Khalyfa et al., 2016; Lapping-Carr et al., 2017). However, some of the small EVs contain marker proteins implying origin from platelets, white blood cells, and endothelial cells. EVs that derive from monocytes are more abundant in SCD subjects than in controls (Lapping-Carr et al., 2017). Even when at a healthy baseline, the abundance of circulating small EVs was greater in individuals with SCD than in control subjects without the disease (Lapping-Carr et al., 2017). However, the number of plasma small EVs did not differ significantly among patients at baseline nor did they differ within the same individual whether obtained at baseline vs. during an episode of acute chest syndrome (ACS; Lapping-Carr et al., 2017, 2020). There are differences in microRNA profiles of the EVs from healthy controls vs. mild vs. severe SCD subjects (Khalyfa et al., 2016).

**Figure 1:**
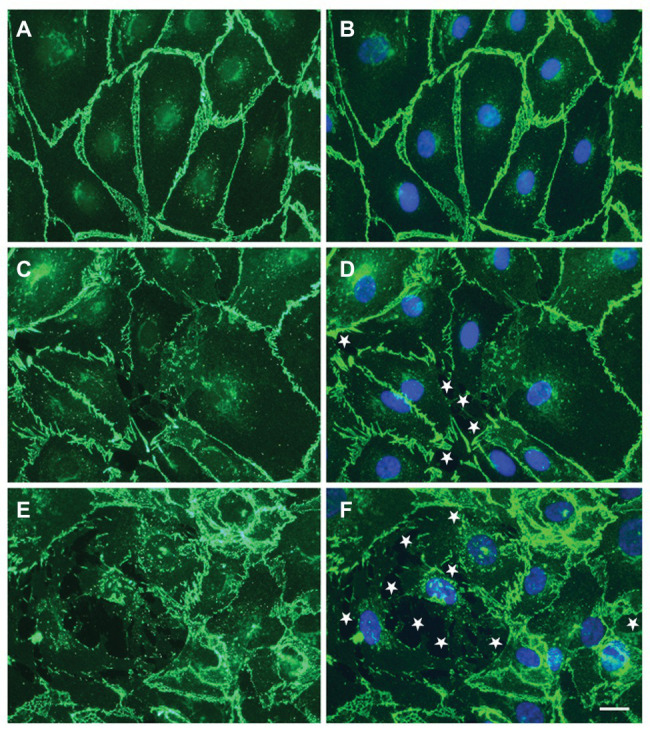
Circulating small extracellular vesicles (EVs) from sickle cell patients cause damage to endothelial cell monolayers. **(A–F)** representative photomicrographs show the localization of VE-cadherin (green) and nuclei (blue in **B,D,F**) in endothelial cells 48 h following treatment with no EVs **(A,B)** or EVs from a subject with sickle cell disease (SCD) purified by precipitation **(C,D)** or by size exclusion chromatography **(E,F)**. White stars indicate spaces between cells. Scale bar is 20 μm. In the examples shown, the monolayer disruption was 0% **(A,B)**, 1.9% **(C,D)**, or 7.5% **(E,F)**. This figure contains different examples illustrating the observations in our previous publication (Lapping-Carr et al., 2017, 2020).

Because of their roles in other vasculopathies, we considered that circulating EVs might contribute to the vascular damage of SCD. We tested this hypothesis by applying small EVs isolated from plasma to monolayers formed of cultured microvascular endothelial cells. Monolayer integrity was studied physiologically (by Electric Cell-substrate Impedance Sensing; Khalyfa et al., 2016; Lapping-Carr et al., 2017) or by microscopy (Lapping-Carr et al., 2017, 2020). Electric Cell-substrate Impedance Sensing (ECIS) studies showed that small EVs isolated at baseline from subjects with a history of ACS caused greater monolayer disruption than was caused by EVs from patients with no history of ACS. This endothelial disruption can be visualized as the opening of spaces between cells in the monolayer (as illustrated in Figure 1). Moreover, small EVs isolated from the same patient during an episode of ACS cause substantially more monolayer disruption, detected as opening of spaces between cells, and reductions of intercellular junction proteins (including VE-cadherin and ZO-1; Khalyfa et al., 2016; Lapping-Carr et al., 2020).

**Figure 2:**
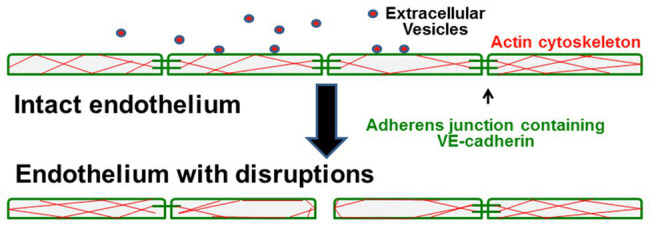
Model illustrates how circulating small EVs cause endothelial damage in sickle cell patients. In control patients or healthy patients with SCD, the endothelial cells form tight monolayers. They are held together by adhesive intercellular junctions containing VE-cadherin, and they have filamentous-actin distributed throughout the cells. In patients that develop the vasculopathy of acute chest syndrome, interaction of circulating small EVs with the endothelial cells causes disruption and loss of some adherens junctions, rearrangement of the actin cytoskeleton, and opening of spaces between cells that disrupt monolayer integrity.

These studies have led us to a model regarding how circulating EVs might contribute to the pathophysiology of ACS (and perhaps other vascular complications of SCD; illustrated in Figure 2). Small EVs/exosomes (packaged with unique proteins and nucleic acids, including mRNAs and miRNAs) undergo regulated release and subsequent uptake by endothelial cells. In healthy control subjects and in a subset of individuals with SCD (at baseline), small EVs in the plasma have no detectable effect on the endothelium. However, in some SCD patients (those with a history of ACS at baseline and more so during an ACS episode), circulating small EVs encounter the endothelium. They cause a series of changes including disruption of intercellular junctions, rearrangement of the actin cytoskeleton, and opening of gaps between cells.

## Future Directions

Our model and the recent studies from other groups raise a number of issues and testable questions. What are the qualitative differences between small EVs of patients with or without a history of ACS? What is the cellular source of the EVs that are important in the vasculopathy of SCD? What are the differences between EVs isolated from the same patient at baseline and during an episode of ACS? Are similar differences present in patients with other complications (like vaso-occlusive crises)? Are these differences due to differing contents of microRNAs that influence gene expression in the target endothelial cells or due to protein components that stimulate inflammation? Can we identify specific EV components (like microRNAs or protein components) that mediate endothelial changes? Can we use them as biomarkers to identify patients at increased risk or as novel therapeutic targets?

## Author Contributions

All authors participated in writing and editing the manuscript. GL-C developed the biobank of specimens. JG and YM performed the experiments. All authors contributed to the article and approved the submitted version.

### Conflict of Interest

The authors declare that the research was conducted in the absence of any commercial or financial relationships that could be construed as a potential conflict of interest.

## References

[ref1] Agouti I. Cointe S. Robert S. Judicone C. Loundou A. Driss F.. (2015). Platelet and not erythrocyte microparticles are procoagulant in transfused thalassaemia major patients. Br. J. Haematol. 171, 615–624. 10.1111/bjh.13609, PMID: 26205481

[ref2] Alaarg A. Schiffelers R. M. van Solinge W. W. van Wijk R. (2013). Red blood cell vesiculation in hereditary hemolytic anemia. Front. Physiol. 4:365. 10.3389/fphys.2013.00365, PMID: 24379786 PMC3862113

[ref3] Allan D. Limbrick A. R. Thomas P. Westerman M. P. (1981). Microvesicles from sickle erythrocytes and their relation to irreversible sickling. Br. J. Haematol. 47, 383–390. 10.1111/j.1365-2141.1981.tb02805.x, PMID: 6779851

[ref4] Allan D. Limbrick A. R. Thomas P. Westerman M. P. (1982). Release of spectrin-free spicules on reoxygenation of sickled erythrocytes. Nature 295, 612–613. 10.1038/295612a0, PMID: 7057919

[ref5] Almizraq R. J. Norris P. J. Inglis H. Menocha S. Wirtz M. R. Juffermans N.. (2018). Blood manufacturing methods affect red blood cell product characteristics and immunomodulatory activity. Blood Adv. 2, 2296–2306. 10.1182/bloodadvances.2018021931, PMID: 30217795 PMC6156888

[ref6] Arvidsson I. Ståhl A. -L. Hedström M. M. Kristoffersson A. -C. Rylander C. Westman J. S.. (2015). Shiga toxin-induced complement-mediated hemolysis and release of complement-coated red blood cell-derived microvesicles in hemolytic uremic syndrome. J. Immunol. 194, 2309–2318. 10.4049/jimmunol.1402470, PMID: 25637016

[ref7] Barry O. P. Praticò D. Savani R. C. FitzGerald G. A. (1998). Modulation of monocyte-endothelial cell interactions by platelet microparticles. J. Clin. Invest. 102, 136–144. 10.1172/JCI2592, PMID: 9649567 PMC509075

[ref8] Bevers E. M. Wiedmer T. Comfurius P. Shattil S. J. Weiss H. J. Zwaal R. F.. (1992). Defective Ca(2+)-induced microvesiculation and deficient expression of procoagulant activity in erythrocytes from a patient with a bleeding disorder: a study of the red blood cells of Scott syndrome. Blood 79, 380–388. 10.1182/blood.V79.2.380.380, PMID: 1730083

[ref9] Bi S. Wang C. Jin Y. Lv Z. Xing X. Lu Q. (2015). Correlation between serum exosome derived miR-208a and acute coronary syndrome. Int. J. Clin. Exp. Med. 8, 4275–4280. PMID: 26064341 PMC4443175

[ref10] Boilard E. Duchez A. -C. Brisson A. (2015). The diversity of platelet microparticles. Curr. Opin. Hematol. 22, 437–444. 10.1097/MOH.0000000000000166, PMID: 26214207

[ref11] Brodsky S. V. Zhang F. Nasjletti A. Goligorsky M. S. (2004). Endothelium-derived microparticles impair endothelial function in vitro. Am. J. Physiol. Heart Circ. Physiol. 286, H1910–H1915. 10.1152/ajpheart.01172.2003, PMID: 15072974

[ref12] Camus S. M. De Moraes J. A. Bonnin P. Abbyad P. Le Jeune S. Lionnet F.. (2015). Circulating cell membrane microparticles transfer heme to endothelial cells and trigger vasoocclusions in sickle cell disease. Blood 125, 3805–3814. 10.1182/blood-2014-07-589283, PMID: 25827830 PMC4490297

[ref13] Cocucci E. Meldolesi J. (2015). Ectosomes and exosomes: shedding the confusion between extracellular vesicles. Trends Cell Biol. 25, 364–372. 10.1016/j.tcb.2015.01.004, PMID: 25683921

[ref14] Colombo M. Raposo G. Théry C. (2014). Biogenesis, secretion, and intercellular interactions of exosomes and other extracellular vesicles. Annu. Rev. Cell Dev. Biol. 30, 255–289. 10.1146/annurev-cellbio-101512-122326, PMID: 25288114

[ref15] Crawford N. (1971). The presence of contractile proteins in platelet microparticles isolated from human and animal platelet-free plasma. Br. J. Haematol. 21, 53–69. 10.1111/j.1365-2141.1971.tb03416.x, PMID: 4254312

[ref16] Feng B. Chen Y. Luo Y. Chen M. Li X. Ni Y. (2010). Circulating level of microparticles and their correlation with arterial elasticity and endothelium-dependent dilation in patients with type 2 diabetes mellitus. Atherosclerosis 208, 264–269. 10.1016/j.atherosclerosis.2009.06.037, PMID: 19674745

[ref17] Freitas Leal J. K. Preijers F. Brock R. Adjobo-Hermans M. Bosman G. (2019). Red blood cell homeostasis and altered vesicle formation in patients with paroxysmal nocturnal hemoglobinuria. Front. Physiol. 10:578. 10.3389/fphys.2019.00578, PMID: 31156458 PMC6529780

[ref18] Garnier Y. Ferdinand S. Garnier M. Cita K. -C. Hierso R. Claes A.. (2020). Plasma microparticles of sickle patients during crisis or taking hydroxyurea modify endothelium inflammatory properties. Blood 136, 247–256. 10.1182/blood.2020004853, PMID: 32285120

[ref19] Giebel B. Kordelas L. Börger V. (2017). Clinical potential of mesenchymal stem/stromal cell-derived extracellular vesicles. Stem Cell Investig. 4:84. 10.21037/sci.2017.09.06, PMID: 29167805 PMC5676188

[ref20] Hebbel R. P. (2014). Ischemia-reperfusion injury in sickle cell anemia: relationship to acute chest syndrome, endothelial dysfunction, arterial vasculopathy, and inflammatory pain. Hematol. Oncol. Clin. North Am. 28, 181–198. 10.1016/j.hoc.2013.11.005, PMID: 24589261

[ref21] Hebbel R. P. Belcher J. D. Vercellotti G. M. (2020). The multifaceted role of ischemia/reperfusion in sickle cell anemia. J. Clin. Invest. 130, 1062–1072. 10.1172/JCI133639, PMID: 32118586 PMC7269579

[ref22] Hebbel R. P. Key N. S. (2016). Microparticles in sickle cell anaemia: promise and pitfalls. Br. J. Haematol. 174, 16–29. 10.1111/bjh.14112, PMID: 27136195

[ref23] Hebbel R. P. Yamada O. Moldow C. F. Jacob H. S. White J. G. Eaton J. W. (1980). Abnormal adherence of sickle erythrocytes to cultured vascular endothelium: possible mechanism for microvascular occlusion in sickle cell disease. J. Clin. Invest. 65, 154–160. 10.1172/JCI109646, PMID: 7350195 PMC371350

[ref24] Hoover R. Rubin R. Wise G. Warren R. (1979). Adhesion of normal and sickle erythrocytes to endothelial monolayer cultures. Blood 54, 872–876. 10.1182/blood.V54.4.872.872, PMID: 476304

[ref25] Hoppe C. C. (2014). Inflammatory mediators of endothelial injury in sickle cell disease. Hematol. Oncol. Clin. North Am. 28, 265–286. 10.1016/j.hoc.2013.11.006, PMID: 24589266

[ref26] Jansen F. Nickenig G. Werner N. (2017). Extracellular vesicles in cardiovascular disease: potential applications in diagnosis, prognosis, and epidemiology. Circ. Res. 120, 1649–1657. 10.1161/CIRCRESAHA.117.310752, PMID: 28495995

[ref27] Jansen F. Yang X. Proebsting S. Hoelscher M. Przybilla D. Baumann K.. (2014). MicroRNA expression in circulating microvesicles predicts cardiovascular events in patients with coronary artery disease. J. Am. Heart Assoc. 3:e001249. 10.1161/JAHA.114.001249, PMID: 25349183 PMC4338711

[ref28] Johnstone R. M. Adam M. Hammond J. R. Orr L. Turbide C. (1987). Vesicle formation during reticulocyte maturation. Association of plasma membrane activities with released vesicles (exosomes). J. Biol. Chem. 262, 9412–9420. PMID: 3597417

[ref29] Kato G. J. Steinberg M. H. Gladwin M. T. (2017). Intravascular hemolysis and the pathophysiology of sickle cell disease. J. Clin. Invest. 127, 750–760. 10.1172/JCI89741, PMID: 28248201 PMC5330745

[ref30] Khalyfa A. Khalyfa A. A. Akbarpour M. Connes P. Romana M. Lapping-Carr G.. (2016). Extracellular microvesicle microRNAs in children with sickle cell anaemia with divergent clinical phenotypes. Br. J. Haematol. 174, 786–798. 10.1111/bjh.14104, PMID: 27161653

[ref31] Kidd L. Geddings J. Hisada Y. Sueda M. Concannon T. Nichols T.. (2015). Procoagulant microparticles in dogs with immune-mediated hemolytic anemia. J. Vet. Intern. Med. 29, 908–916. 10.1111/jvim.12583, PMID: 25871966 PMC4895429

[ref32] Klaihmon P. Phongpao K. Kheansaard W. Noulsri E. Khuhapinant A. Fucharoen S.. (2017a). Microparticles from splenectomized β-thalassemia/HbE patients play roles on procoagulant activities with thrombotic potential. Ann. Hematol. 96, 189–198. 10.1007/s00277-016-2885-6, PMID: 27900452

[ref33] Klaihmon P. Vimonpatranon S. Noulsri E. Lertthammakiat S. Anurathapan U. Sirachainan N.. (2017b). Normalized levels of red blood cells expressing phosphatidylserine, their microparticles, and activated platelets in young patients with β-thalassemia following bone marrow transplantation. Ann. Hematol. 96, 1741–1747. 10.1007/s00277-017-3070-2, PMID: 28748286

[ref34] Kozuma Y. Sawahata Y. Takei Y. Chiba S. Ninomiya H. (2011). Procoagulant properties of microparticles released from red blood cells in paroxysmal nocturnal haemoglobinuria. Br. J. Haematol. 152, 631–639. 10.1111/j.1365-2141.2010.08505.x, PMID: 21241275

[ref35] Kuo W. P. Tigges J. C. Toxavidis V. Ghiran I. (2017). Red blood cells: a source of extracellular vesicles. Methods Mol. Biol. 1660, 15–22. 10.1007/978-1-4939-7253-1_2, PMID: 28828644

[ref36] Lapping-Carr G. Gemel J. Mao Y. Sparks G. Harrington M. Peddinti R.. (2020). Circulating extracellular vesicles from patients with acute chest syndrome disrupt adherens junctions between endothelial cells. Pediatr. Res. 10.1038/s41390-020-0923-5 [Epub ahead of print], PMID: 32454519 PMC8261277

[ref37] Lapping-Carr G. Khalyfa A. Rangel S. Darlington W. Beyer E. C. Peddinti R.. (2017). Exosomes contribute to endothelial integrity and acute chest syndrome risk: preliminary findings. Pediatr. Pulmonol. 52, 1478–1485. 10.1002/ppul.23698, PMID: 28486752 PMC5653417

[ref38] Mahfoudhi E. Lecluse Y. Driss F. Abbes S. Flaujac C. Garçon L. (2012). Red cells exchanges in sickle cells disease lead to a selective reduction of erythrocytes-derived blood microparticles. Br. J. Haematol. 156, 545–547. 10.1111/j.1365-2141.2011.08897.x, PMID: 21988211

[ref39] Mantel P. -Y. Hoang A. N. Goldowitz I. Potashnikova D. Hamza B. Vorobjev I.. (2013). Malaria-infected erythrocyte-derived microvesicles mediate cellular communication within the parasite population and with the host immune system. Cell Host Microbe 13, 521–534. 10.1016/j.chom.2013.04.009, PMID: 23684304 PMC3687518

[ref40] Meldolesi J. (2018). Exosomes and ectosomes in intercellular communication. Curr. Biol. 28, R435–R444. 10.1016/j.cub.2018.01.059, PMID: 29689228

[ref41] Merle N. S. Grunenwald A. Rajaratnam H. Gnemmi V. Frimat M. Figueres M. -L.. (2018). Intravascular hemolysis activates complement via cell-free heme and heme-loaded microvesicles. JCI Insight 3:e96910. 10.1172/jci.insight.96910, PMID: 29925688 PMC6124427

[ref42] Mezentsev A. Merks R. M. H. O’Riordan E. Chen J. Mendelev N. Goligorsky M. S.. (2005). Endothelial microparticles affect angiogenesis in vitro: role of oxidative stress. Am. J. Physiol. Heart Circ. Physiol. 289, H1106–H1114. 10.1152/ajpheart.00265.2005, PMID: 15879485

[ref43] Morel O. Jesel L. Freyssinet J. -M. Toti F. (2011). Cellular mechanisms underlying the formation of circulating microparticles. Arterioscler. Thromb. Vasc. Biol. 31, 15–26. 10.1161/ATVBAHA.109.200956, PMID: 21160064

[ref44] Nebor D. Bowers A. Connes P. Hardy-Dessources M. -D. Knight-Madden J. Cumming V.. (2014). Plasma concentration of platelet-derived microparticles is related to painful vaso-occlusive phenotype severity in sickle cell anemia. PLoS One 9:e87243. 10.1371/journal.pone.0087243, PMID: 24475257 PMC3901744

[ref45] Nébor D. Romana M. Santiago R. Vachiery N. Picot J. Broquere C.. (2013). Fetal hemoglobin and hydroxycarbamide moduate both plasma concentration and cellular origin of circulating microparticles in sickle cell anemia children. Haematologica 98, 862–867. 10.3324/haematol.2012.073619, PMID: 23403312 PMC3669440

[ref46] Nguyen D. B. Ly T. B. T. Wesseling M. C. Hittinger M. Torge A. Devitt A.. (2016). Characterization of microvesicles released from human red blood cells. Cell. Physiol. Biochem. 38, 1085–1099. 10.1159/000443059, PMID: 26938586

[ref47] Ninomiya H. Kawashima Y. Hasegawa Y. Nagasawa T. (1999). Complement-induced procoagulant alteration of red blood cell membranes with microvesicle formation in paroxysmal nocturnal haemoglobinuria (PNH): implication for thrombogenesis in PNH. Br. J. Haematol. 106, 224–231. 10.1046/j.1365-2141.1999.01483.x, PMID: 10444191

[ref48] Nomura S. Shimizu M. (2015). Clinical significance of procoagulant microparticles. J. Intensive Care 3:2. 10.1186/s40560-014-0066-z, PMID: 25705427 PMC4336124

[ref49] Oggero S. Austin-Williams S. Norling L. V. (2019). The contrasting role of extracellular vesicles in vascular inflammation and tissue repair. Front. Pharmacol. 10:1479. 10.3389/fphar.2019.01479, PMID: 31920664 PMC6928593

[ref50] Olatunya O. S. Lanaro C. Longhini A. L. Penteado C. F. F. Fertrin K. Y. Adekile A.. (2019). Red blood cells microparticles are associated with hemolysis markers and may contribute to clinical events among sickle cell disease patients. Ann. Hematol. 98, 2507–2521. 10.1007/s00277-019-03792-x, PMID: 31493004

[ref51] Ovchynnikova E. Aglialoro F. von Lindern M. van den Akker E. (2018). The shape shifting story of reticulocyte maturation. Front. Physiol. 9:829. 10.3389/fphys.2018.00829, PMID: 30050448 PMC6050374

[ref52] Owens A. P. Mackman N. (2011). Microparticles in hemostasis and thrombosis. Circ. Res. 108, 1284–1297. 10.1161/CIRCRESAHA.110.233056, PMID: 21566224 PMC3144708

[ref53] Padilla F. Bromberg P. A. Jensen W. N. (1973). The sickle-unsickle cycle: a cause of cell fragmentation leading to permanently deformed cells. Blood 41, 653–660. 10.1182/blood.V41.5.653.653, PMID: 4694082

[ref54] Pattanapanyasat K. Gonwong S. Chaichompoo P. Noulsri E. Lerdwana S. Sukapirom K.. (2007). Activated platelet-derived microparticles in thalassaemia. Br. J. Haematol. 136, 462–471. 10.1111/j.1365-2141.2006.06449.x, PMID: 17278261

[ref55] Ridger V. C. Boulanger C. M. Angelillo-Scherrer A. Badimon L. Blanc-Brude O. Bochaton-Piallat M. -L.. (2017). Microvesicles in vascular homeostasis and diseases. Position Paper of the European Society of Cardiology (ESC) Working Group on Atherosclerosis and Vascular Biology. Thromb. Haemost. 117, 1296–1316. 10.1160/TH16-12-0943, PMID: 28569921

[ref56] Said A. S. Doctor A. (2017). Influence of red blood cell-derived microparticles upon vasoregulation. Blood Transfus. 15, 522–534. 10.2450/2017.0353-16, PMID: 28686154 PMC5649961

[ref57] Shet A. S. Aras O. Gupta K. Hass M. J. Rausch D. J. Saba N.. (2003). Sickle blood contains tissue factor-positive microparticles derived from endothelial cells and monocytes. Blood 102, 2678–2683. 10.1182/blood-2003-03-0693, PMID: 12805058

[ref58] Sloand E. M. Maciejewski J. P. Dunn D. Moss J. Brewer B. Kirby M.. (1998). Correction of the PNH defect by GPI-anchored protein transfer. Blood 92, 4439–4445. 10.1182/blood.V92.11.4439, PMID: 9834251

[ref59] Sundd P. Gladwin M. T. Novelli E. M. (2019). Pathophysiology of sickle cell disease. Annu. Rev. Pathol. 14, 263–292. 10.1146/annurev-pathmechdis-012418-012838, PMID: 30332562 PMC7053558

[ref60] Tang N. Sun B. Gupta A. Rempel H. Pulliam L. (2016). Monocyte exosomes induce adhesion molecules and cytokines via activation of NF-κB in endothelial cells. FASEB J. 30, 3097–3106. 10.1096/fj.201600368RR, PMID: 27226520 PMC5001509

[ref61] Tantawy A. A. G. Adly A. A. M. Ismail E. A. R. Habeeb N. M. Farouk A. (2013). Circulating platelet and erythrocyte microparticles in young children and adolescents with sickle cell disease: relation to cardiovascular complications. Platelets 24, 605–614. 10.3109/09537104.2012.749397, PMID: 23249216

[ref62] Teruel-Montoya R. Luengo-Gil G. Vallejo F. Yuste J. E. Bohdan N. García-Barberá N.. (2019). Differential miRNA expression profile and proteome in plasma exosomes from patients with paroxysmal nocturnal hemoglobinuria. Sci. Rep. 9:3611. 10.1038/s41598-019-40453-5, PMID: 30837665 PMC6401143

[ref63] Théry C. Witwer K. W. Aikawa E. Alcaraz M. J. Anderson J. D. Andriantsitohaina R.. (2018). Minimal information for studies of extracellular vesicles 2018 (MISEV2018): a position statement of the International Society for Extracellular Vesicles and update of the MISEV2014 guidelines. J. Extracell. Vesicles 7:1535750. 10.1080/20013078.2018.1535750, PMID: 30637094 PMC6322352

[ref64] Vallejo F. Yuste J. E. Teruel-Montoya R. Luengo-Gil G. Bohdan N. Espín S.. (2019). First exploratory study on the metabolome from plasma exosomes in patients with paroxysmal nocturnal hemoglobinuria. Thromb. Res. 183, 80–85. 10.1016/j.thromres.2019.10.001, PMID: 31671376

[ref65] van Beers E. J. Schaap M. C. L. Berckmans R. J. Nieuwland R. Sturk A. van Doormaal F. F.. (2009). Circulating erythrocyte-derived microparticles are associated with coagulation activation in sickle cell disease. Haematologica 94, 1513–1519. 10.3324/haematol.2009.008938, PMID: 19815831 PMC2770961

[ref66] Vats R. Brzoska T. Bennewitz M. F. Jimenez M. A. Pradhan-Sundd T. Tutuncuoglu E.. (2020). Platelet extracellular vesicles drive inflammasome-IL-1β-dependent lung injury in sickle cell disease. Am. J. Respir. Crit. Care Med. 201, 33–46. 10.1164/rccm.201807-1370OC, PMID: 31498653 PMC6938158

[ref67] Weitz I. C. Razavi P. Rochanda L. Zwicker J. Furie B. Manly D.. (2012). Eculizumab therapy results in rapid and sustained decreases in markers of thrombin generation and inflammation in patients with PNH independent of its effects on hemolysis and microparticle formation. Thromb. Res. 130, 361–368. 10.1016/j.thromres.2012.04.001, PMID: 22542362

[ref68] Westerman M. Pizzey A. Hirschman J. Cerino M. Weil-Weiner Y. Ramotar P.. (2008). Microvesicles in haemoglobinopathies offer insights into mechanisms of hypercoagulability, haemolysis and the effects of therapy. Br. J. Haematol. 142, 126–135. 10.1111/j.1365-2141.2008.07155.x, PMID: 18422994

[ref69] Westerman M. Porter J. B. (2016). Red blood cell-derived microparticles: an overview. Blood Cells Mol. Dis. 59, 134–139. 10.1016/j.bcmd.2016.04.003, PMID: 27282583

